# Exploring the Lived Experience, Meaning and Imperatives of Fatherhood: An Interpretative Phenomenological Analysis

**DOI:** 10.5539/gjhs.v8n9p139

**Published:** 2015-10-29

**Authors:** Narges Eskandari, Masoumeh Simbar, Abou Ali Vadadhir, Ahmad Reza Baghestani

**Affiliations:** 1 Department of Midwifery and Reproductive Health, School of Nursing and Midwifery, Shahid Beheshti University of Medical Sciences, Tehran, Iran; 2 Department of Midwifery and Reproductive Health, School of Nursing and Midwifery & Reproductive Endocrinology Research Center, Research Institute for Endocrine Sciences, Shahid Beheshti University of Medical Science, Tehran, Iran; 3 Associate Professor, Department of Anthropology, Faculty of Social Sciences, Tehran University & Fellow of the Social Health Group, The Academy of Medical Sciences of Iran, Tehran, Iran; 4 Department of Biostatistics, School of Paramedical Sciences, Shahid Beheshti University of Medical Sciences, Tehran, Iran

**Keywords:** fatherhood, fathering, imperatives of fathering, interpretative phenomenological analysis, paternal role

## Abstract

**Introduction:**

There have been considerable changes in the concept and meaning of fatherhood in the past few decades and a lot of studies has down in this area, but there is no information about fathering and fatherhood from Iranian perspective, thus present study designed to explore the men’s understanding of fathering and paternal role during their first year of transition to parenthood.

**Method:**

This phenomenological study included accounts of 17 Iranian fathers, who had experienced fathering for the first time. Data was analyzed using the Interpretative Phenomenological Analysis (IPA) approach.

**Results:**

The results reveal that a father is a man who reproduces a child and accepts the responsibility for supporting his family as the fulcrum. A father is a good-tempered, faithful, patient and hardworking man with essential knowledge and proficiency. A father should accept his role as the father. He is also expected to participate actively in dealing with family daily issues, value and promote the health and well-being of his children, and have skills of self-management and self-care.

**Conclusion:**

Iranian fathers not only committed to play their traditional roles and responsibilities, but also welcome new roles such as constantly being with their children and providing emotional support to them.

## 1. Introduction

Human’s life includes consecutive changes and passages from one phase to another and from one situation to another (Van Gennep, 2011). In one of these passages, man is transformed into a father (Cooper, 2005). Stepping into fatherhood is the most important events that can happen to a man because leads to fundamental changes in his identity and life style (Kowlessar, 2012). In this view, understanding the experiences of fathers and their roles in family has attracted the interests of scholars, and they believe that father plays a key role in formation of family and development of child (Halle et al., 2008). Otherwise, during recent decades, expectations from fathers changed and men’s involvement increased in the tasks of the home that became difficult for women to manage homework and their employment duties simultaneously (Hofferth, & Goldscheider, 2010).

Although fatherhood is a common experience for men in the sense of creating a child, but it is not common in the sense of child nurturing. Researchers believe that two patterns of fatherhood are currently dominant worldwide. One rare pattern that is increasingly gaining popularity includes men who considerably take part in the nurture of their child. The second pattern, which is the most prevalent, includes men with minimum participation in nurture of their child or no caretaking (Tazi-Preve, 2009). One of the most valuable advances in the studies of fatherhood is the effort put into defining and developing the concept of father’s participation and determining paternal activities that can influence the lives of children (Marsiglio, 2009; Sarkadi et al., 2008). According to Lamb et al. (1987), involving in the children’s affairs, availability and responsibility are the criteria for participation of fathers referred by fathers in other studies (Mbekenga et al., 2011; Summers et al., 2006; Chin et al., 2011). These criteria influenced subsequent studies related to fatherhood. In this view, Doherty et al., (1998) used the term of “responsible father”. The responsible father is a man who acts responsibly regarding his children and does in the following ways: he waits to make a baby until he is ready to support the child both financially and emotionally, when the child is born he establishes legal paternity, he actively participates in providing physical and emotional care to the child from the pregnancy and continues to financial support of his child after birth.

Resulting some macro sociocultural and techno-scientific changes in the last two decades, the notion of fatherhood has changed and men are expected to participate more actively in nurturing their children (Hofferth, & Goldscheider, 2010). However, there is little research and information about factors influencing the participation of men in raising their children (Cabrera et al., 2000). Researchers also revealed that fathering is not a static and specific practice and affected by sociocultural and personal factors such as motivation, skill, self-confidence, age and maturity of parents, as well as number, age, gender and developmental stage of the child, and social expectations and constructions (Dowd, 2000).

Consistent with the studies in other settings on the globe, Iranian scholars have recently interested in doing research on the role of men in the family structure and reproductive health (Simbar et al., 2011; Simbar et al., 2010; Ozgoli et al., 2000; Vakilian & Keramat, 2012; Mortazavi & Keramat, 2012; Tremayne, 2015; Golian Tehrani et al., 2014). However, no study has addressed the lived experiences of Iranian fathers and their concepts of fatherhood and its requirements using the Interpretative Phenomenological Analysis (IPA). Due to the existence of various and contesting images and faces of contemporary fatherhood in the Islamic Republic of Iran (Tremayne, 2014), results of this study, providing valuable insights and implications for the policy and the public, is a significant endeavor in conceptualizing fatherhood and its imperatives in the distinctive social-cultural context of Iran.

## 2. Materials and Methods

### 2.1 Setting

This study conducted in Qom, the 8^th^ largest city in Iran and the capital of Qom Province. Qom is considered holy by Shi`a, the second largest denomination of Islam and people from different Iranian ethnicity live in this city.

### 2.2 Sample and Recruitment

Fathers recruitment was down in anywhere which was convenient for them (e.g. in health centers or work places). Researcher was present in these places and tried to meet fathers and invite them to participate in the study, if the fathers meet the inclusion criteria. Participants in this study were men, who experienced fathering for first time. In terms of the inclusion criteria, all fathers were healthy and their age was 20 years or over, they were able to speak and understand Persian language and lived with mother of their child and their infant was singleton and healthy. Lack of interest to participate and remain in the study was the exclusion criteria because some fathers couldn’t explain about their fathering experiences or didn`t desire to speak about it.

### 2.3 Procedures

Participants in this phenomenological study were recruited by purposeful sampling strategy to insure that fathers from different socioeconomic positions are recruited in the study. The sampling procedure continued until the data saturation (Streubert, & Carpenter, 2007) which involves sampling to the point at which themes and categories in the data become repetitive and redundant, such that no new information can be gleaned by further data collection (Polit, & Beck, 2008). In sum, 17 fathers were included in this study.

### 2.4 Data Collection

Data were gathered through individual semi-structured qualitative interviews and a demographic characteristic questionnaire. Interview guide was developed using and reviewing the existing literature and counseling with Iranian experts and adjusted following several pilot interviews. An interview guide was provided general guideline on the subject of fatherhood and included theses question: what did you think about fathering? Explain about your fathering experience; what does fathering mean in your view? Further open-ended questions built upon the fathers’ responses. The main scholar also tried to identify and understand the type and quality of the men’s lived experience by observing the non-verbal reactions of fathers when they remembered their experience of fathering. Secondary interviews were also conducted (if needed) to confirm the data and bridge any possible gap in data. The interviews lasted between 20 to 80 minutes each. All interviews were audio taped, transcribed verbatim and analyzed consecutively.

### 2.5 Ethical Consideration

Permission to carry out this study was obtained from Tehran’s Shahid Beheshti University of Medical Sciences Ethical Committee on 24 January 2013 (sbmuz.rec.1394.76) and submitted to the relevant local authorities and the health centers of Qum. Scholars of the study obtained permission of all participants to perform and audio-tape the interviews, formally. To accomplish this, the objectives and methods of the research were briefly explained to the fathers and their dignity, privacy and freedom of actions was respected throughout interviews. The confidentiality of information was guaranteed, as the name and personal information of interviewees was not mentioned in tapes and transcripts.

### 2.6 Data Analysis and Rigors

The transcripts were qualitatively managed and analyzed using IPA as termed by Smith and Osborn (2008) and Smith, Flowers, & Larkin, (2009). In accordance with the IPA process each transcript was read and re-read to enable the scholars to become adequately familiar with the accounts. Through an iterative process, tentative labels were created to capture the essence of the ideas within each interview, and subsequently interrelated and clustered into a number of sub-ordinate themes. Then, shared themes were organized across transcripts and super-ordinate themes formed. To assure the quality and trustworthiness criteria, three researchers regularly discussed their procedure of data analysis and themes generation. Strategies of member checking were used to ensure credibility of research findings. For instance, the manuscripts of interviews were returned to the interviewees and asked to determine whether the transcripts reflected their experiences or not. To ensure transferability, different stages of the study were properly explained and documents of study were provided for auditing. As a final point, qualitative data analysis software, i.e. MAXQDA, was used to enrich and substantiate the coding process and facilitate data analysis phase of the study.

## 3. Results

In total, 17 men experienced the fatherhood for the first times were interviewed. The mean of fathers’ age was 28.4 years (25-31years). They were from different socio-economic classes and were living in urban and rural areas. All fathers were healthy and lived with their healthy wives and children. The demographic characteristics of fathers participating in the study are presented in Table 1.

**Table 1:** Demographic characteristics of the participants in the study

No	Age	Job	Economic status	Education	Ethnicity	Residence	Child Sex	Child age (month)
1	26	Employee	Average	Diploma	Persian	Urban	Girl	8
2	30	Employee	Good	Bachelor	Persian	Urban	Girl	2
3	29	Sweeper	Weak	Middle school	Azerbaijani	Urban	Boy	7
4	28	Cloth production	Average	Bachelor	Persian	Urban	Boy	2
5	29	Employee	Average	Diploma	Persian	Urban	Girl	12
6	25	Islamic clergy	Good	Religious training	Persian	Urban	Girl	5
7	31	Building painter	Weak	Bachelor	Azerbaijani	Urban	Girl	12
8	26	Islamic clergy	Average	Religious training	Persian	Urban	Boy	11
9	30	Public Relations Officer	Average	Master degree	Gilaki	Urban	Boy	1
10	27	Green space worker	Weak	Fifth grade	Azerbaijani	Urban	Girl	12
11	28	Unemployed	Weak	Illiterate	Persian	Rural	Boy	6
12	30	Building technician	Average	Middle school	Persian	Rural	Boy	10
13	28	Hospital services	Weak	Diploma	Persian	Urban	Girl	12
14	29	Employee	Average	Bachelor	Persian	Urban	Boy	2
15	30	Spring maker	Average	High school	Persian	Rural	Boy	12
16	31	Emergency staff	Average	Associate degree	Persian	Urban	Girl	4-month fetus
17	25	Blacksmith	Weak	Primary school	Persian	Urban	Girl	7-month fetus

Table 2 displays the super- and sub-ordinate themes derived from this qualitative study. Based on the lived experience and understanding of men about the fathering and paternal roles as well as the collected qualitative data, finally 17 themes, 4 sub-ordinate themes and 2 super-ordinate themes were obtained.

**Table 2:** Overview of super-ordinate and sub-ordinate themes derived from the qualitative study

Supra-ordinate themes	Sub-ordinate themes	Themes
**Concept of fatherhood**	Definition of fatherhood	Reproduction of a child
		Responsibility
		The fulcrum of the family
	Rationales for being a father	Response to innate needs
		Desire to survive and continuity
		Social stimulus
**Requirements of fatherhood**	Acquisition of paternal traits	Father as a qualified person proficiency
		Father as a skilled director
		Good-tempered
		Devoted
		Faithful
		Patient and hardworking
	Fulfillment of paternal roles and responsibilities	Accepting the paternal role
		Participating in all affairs of the family
		Ensuring health of the child
		Upbringing the child
		Self-management and self-care

The super-ordinate themes included the concept of fatherhood and requirements of fatherhood and sub-ordinate themes included the definition of fatherhood, rationales for being a father, acquisition of paternal traits, fulfillment of paternal roles and responsibilities that are explained as follows.

### 3.1 Concept of Fatherhood

When fathers’ view about the fatherhood was asked, they used different phrases to describe it. These phrases can be classified into two categories: definition of fatherhood and reasons for becoming a father.

#### 3.1.1 Definition of Fatherhood

Based on the participants’ perception, three main definitions of fatherhood were revealed; a father is a man who becomes a father through childbirth, He acts as the fulcrum for the family and accepts new responsibilities regarding his child and wife.

Reproduction of a child. Most fathers believed to this traditional and common perspective that when a man’s child is born, he owns the title of “father”. In fact, childbirth means becoming a parent, inheritance of genes to the next generation, and having the guardianship and ownership of child. As one of the fathers noted,

“Now based on the marital relationship, person who owned a child, the title of father can be given to him that means he has a child.” (Father 14)

Responsibility. Nearly all participants considered fatherhood equivalent to responsibility. That means when they were asked what the mean of fatherhood is, they would quickly say fatherhood means responsibility and mentioned to enumerate a father’s responsibilities. According to the fathers, these responsibilities included all of the aspects of children’s life. A number of fathers stated,

“He is responsible to the child, responsible for all things related to him, like spiritual, mental and economic needs…” (Father 17).

“A man who may have child but may reject responsibilities, cannot be called a father” (Father 6).

The fulcrum of the family. According to the participants, father is the main pillar and the fulcrum of the family because he plays the main role in supporting the family, and decisions making. The participants mentioned some of ways for supporting family including meeting the physical and mental needs of their wife and child, planning for future, and ensuring the welfare and comfort of their families. Fathers’ capability to meet the needs of family is so important that some people consider it as a criterion for assessing their performance and believed that if a father cannot meet the needs of his family, he is not a competent father. For most fathers, economic and financial needs form an important part of family needs, thus they set it in the first of their responsibilities. As a father added,

“I put it in one sentence: only be able to meet the needs of his child. That’s it” (Father 13).

Most fathers stated that fathers are obliged to provide for the comfort and peace of their wife and child. Fathers also considered themselves as emotional supporter for their families and responsible for the grief and joy as well as the tears and laughter of their wife and child. As a father noted,

“A responsible father is responsible for the laughter and tears of his child or mother of child, accepts all responsibilities of them” (Father 4).

However, one of the fathers believed that acting as the fulcrum for the family is not related to the presence of child.

“Regarding role of the father in the family, it can be said that… he is the core of the family… whether he has a child or not…. Hence, I believe in our society, father forms one of the main pillars of the family” (Father 9).

#### 3.1.2 Rationales for Being a Father

Some participants considered fatherhood as a natural and innate need and believed that factors such as willingness for survival and continuity, and social stimulus reinforce their interest in becoming a father.

Response to innate needs. A number of men consider fatherhood to be an innate and natural need that is put inside all men. This innate willingness guides men toward fathering. As a participant father stated,

“Everyone desires to be a father. I personally wanted to know the taste of fatherhood” (Father 12).

Desire to survive and continuity. The desire for survival has root in human innate and the presence of child ensures fathers that family name and property will maintain after their death. A father said,

“In all society, every family should create a child after 1 to 2 years depending on its conditions or function, to contribute to the survival and continuity of human generation” (Father 9).

Social stimulus. When a young man sees the relationship between fathers and their children, he envies them and wishes to have such a fathering position. It seems he feels something lacks inside when he compares himself to fathers. Moreover, as a result of the pressure by the relatives and significant others on young couple for not having a child, the husband might feel dubious about his own fertility and may decide to produce a child to prove his health and decrease the pressure by relatives. As a father explained,

“When you come and say to man: ‘don’t you want to be a father?’ I say myself ‘what he means? Does he mean there is something wrong with me? …” (Father 13).

### 3.2 Requirements of Fatherhood

The participants of this study believed that they should have requirements as fathers which included the acquisition of the fathering qualities and playing role of the fathering and fulfilling fathering responsibilities. Acquiring the capability for developing the traits and fulfilling fatherhood responsibilities are the requirements for fathering according to the fathers’ statements.

#### 3.2.1 Acquisition of Paternal Traits

The participants stated that fathers should be skilled directors, good-tempered, devoted, lenient, faithful, patient, hardworking, and knowledgeable about their fatherhood duties.

Father as a qualified person. According to men, fathers should face hardships of life and gain adequate experiences in different stages of life, in order to deal with and overcome all problems associated with fatherhood. Moreover, fathers should have sufficient knowledge about child rearing and nurturing and get ready to answer the needs and questions of their children.

“A father is someone who understands life and has experienced complications and worries of life and its ups and downs” (Father 11).

Father as a skilled director. Almost half of the precipitants referred to management as an important feature of fathers. One of the fathers referred to prioritization as an important management skill and commented that a qualified father should assess and prioritize his family needs. In order to obtain satisfactory results, fathers should consult their family members, think deeply and make careful decisions. The fathers noted that their decisions would influence future status of their family. Hence, they should first consider benefits of the family.

“He has to think deeply and make more careful decisions for the life which he is responsible for” (Father 14).

Good-tempered. A good-tempered father means that he can control his wrath and does not transfer the outdoor problems or anxieties to his family environment. Such a father treats his wife and children properly and does not hurt or annoy them. As a father described it in this way,

“When I am angry I try to keep my anger to myself. I control myself and try to calm down to be able to cope with my wife and child peacefully” (Father 11).

Devoted. Forgiving and sacrificing are capabilities that every father should necessarily possess because sometimes it is necessary for a father to ignore his own interests and benefits for the sake of his family and child. As a father explained,

“He does not think just about himself anymore as he does not belong to himself, he almost thinks about his child” (Father 16).

Faithful. One father believed that fathers should be committed to religious and spiritual principles and shall apply those to their life because religious principles are guides and ethic guarantees for fathers. Religious decrees also provide the framework for true raising and training of children.

“Should to make licit money and bring it to his family, be faithful and say his prayers.” (Father 10).

Patient and hardworking. A father has better know that raising children is not a simple duty and he will face ups and downs as a father. Therefore, he should be ready to face such hardships and does his best to fulfill his duties. Fathers considered the degree of their efforts as a criterion for assessing their capabilities and dutifulness. They believed that although sometimes they cannot bear the heavy burdens of their obligations and may fail to attain their goals, they proud of themselves because know that they did not neglect their duties.

“If he had tried since he can, he is a good father by himself. Even he fails, due to existing conditions or barriers, because there are some things, there are obstacles, if it happens; he knows that made his effort.” (Father 1).

#### 3.2.2 Fulfillment of Paternal Roles and Responsibilities

Men enumerated the following roles and responsibilities as their fathering duties: Fathers should accept themselves as father; participate in family affairs, value the health and upbringing of their child, and have self-management and self-care skills.

Accepting the paternal role. Accepting the paternal role means understanding the presence of child in their life and one’s role as a father. According to the majority of participants, a real father should first accept he is a father. Then He has to adjust his behavior and functions in accordance with his paternal role. A father should pay attention to the presence of child in his life and accept himself as the responsible one for the child life.

“A good father is someone who … first of all, accepts that he has become a father.” (Father 6).

Participating in all affairs of the family. Every father is responsible to dedicate his time to his family and plan to be present in the family. Father`s time is allocated in two ways to the family: he should spend his spare time with his family; he shold spend his time to supply the welfare and comfort for his family.

“A father is someone who spends most of his time with his family and on making a livelihood for his family.” (Father 7).

To achieve this aim, fathers should revised their daily schedules and allocate a time to establish relations with children and handle their affairs in spite of fatigue and occupation. Fathers not only emphasized on the necessity of allocating their time to family but also believed that they have to plan on the entertainment and recreation of their families to provide happiness and joy.

Most fathers stated that they should participate actively in their family affairs. This participation includes taking part in raising children and doing the housework. As a father said,

“Putting her to sleep and feeding her with milk… since my child feeds on infant formula I help my wife feed her” (Father 10).

One of the fathers believed that reducing expectations from wife is one of most significant contributions of men as this approach to marital life reduces the burden on the wife. As he said,

“Well, first I reduce my expectations. When I reduce my expectations, it is one of the biggest contributions” (Father 5).

Although fathers believed it is their duty to take part in family affairs, most of them expressed that they were not considerably participating in raising the child. As they believed housekeeping and child rising are wifely duties and father participation is a form of meddling with the duties of their wives.

“Look! Some activities are just for mothers. For instance, washing clothes are for mother, changing children’s clothes is for mother, taking children to the toilet, bathing children are all for mothers. …. I don’t do special work. …. However, we take the baby out, we do it …., but most of the work is done by the mother and I try not to meddle in her duties” (Father 13).

Fathers believed that existence of a proper fatherhood model, having a positive attitude toward participation in raising child, acquiring skills for caring children, increasing the time spent in house, and reducing concerns are factors that provide context for participation of fathers.

Ensuring health of the child. In order to ensure the health of their child, fathers try to provide hygienic and healthy materials and medical care to their child. In spite of financial constraints, some fathers insisted on provide money to use of nongovernmental services, as they did not fully trust the quality of the governmental medical services.

“I earn the money from any possible source, take the baby, no one day later, to examine his weight, body, hearing, and vision” (Father 3).

Upbringing the child. Child upbringing is an important responsibility that was referred approximately by all fathers. They used the following methods to attain this goal: becoming appropriate role models for child; playing the role of a good consultant for child; paying attention to the religious upbringing of child; selecting proper companions for the family; establishing a firm intimate connection with child; allocating time and energy to upbringing the child; preparing child for social life; and acquiring knowledge about child upbringing. As a participant highlighted,

“I extremely pay attention to the type of conversation with child, or we try to talk carefully at home. Up to now I think my child senses many things. I don’t mean he understands them but he feels. So we try to be very cautious in our talking” (Father 4).

Self-management and self-care. Most fathers stressed that they should be cautious for their behaviors and words as a father, improve defects and shortages, correct past mistakes, have rational self-expectations and define criteria for self. These capabilities make fathers able to act as a good father and proper role models for their child. Fathers also had better take care of their physical health as they are the protector of their families and any damages to their health would lead to problems for the family. As a number of fathers declared,

“He should be cautious about some things. For example, he should not ride motorcycles as he has a duty to constantly ask this question from himself if I go and an accident occurs for me then what happens for my child” (Father 16).

## 4. Discussion

This study was the first study carried out in Iran to clarify the concept of fatherhood based on men’s understanding and lived experience of fathering using the IPA methodology. Results of this study revealed that from the viewpoint of Iranian new fathers, fathering can be categorized in two major themes: concept of fatherhood and requirements of fatherhood.

The men participating in this study believed that a father is a man who inherits a neonate through child birth. He acts as the fulcrum for the family and accepts new responsibilities to his child and wife. As mentioned, the definition of fathering provided by Iranian men was a combination of the biological and social father concepts (Barenski, 2010). Also, this is in line with the definition of productive father. A productive father is a man who cares and produces new generations and expected to be present both physically and practically in the family to provide support and care to his children (Hawkins, & Dollahite, 1997). According to Iranian men, the father is the main pillar and main member of the family who is responsible for making final decisions and acting as a strong and constant support for the family. This definition is roughly original as it was not reported in the other studies. It seems in other countries, fathers have lost their place as the main supporter due to the employment of women and prevalence of single-parent families.

Iranian fathers referred to traditional duties (e.g. meeting financial needs, supporting families and child rearing) and modern duties (presence, participation and emotional support) (Pleck, & Pleck, 1997), in explaining their responsibilities. This reveals that Iranian fathers accept modern roles without leaving traditional roles. They also demonstrate the concept of the participating father in their action, as previously described by Lamb et al. (1987), because they referred to concepts such as participation, availability and responsibility referred by fathers in other studies (Mbekenga, Lugina, Christensson, & Olsson, 2011; Summers, Boller, Schiffman, & Raikes, 2006; Chin et al., 2011).

During recent decades, following changes in the globalized world, the concept of fatherhood has changed and men are expected to participate more actively in child nurturing (Barenski, 2010). However, results of this study and some other researches showed that fathers slightly participate in family affairs and child nurturing (Mortazavi, & Keramat, 2012). Researchers believe that cultural beliefs, gender roles and stereotypes, and social norms can influence the performance of men in the family (Simbar et al., 2009) and inflexible employment schedules, strong gender-role stereotypes, and legal divorce settlements are obstacles that prevent fathers from participating in family affairs and child rearing (Hofferth & Goldscheider, 2015). Father involvement also is strongly influenced by the mother, both in terms of gatekeeping and modeling (Hofferth & Goldscheider, 2015). In this study, availability of a proper fatherhood model, a positive attitude toward participation in child rearing, gaining skills for taking care of children, increasing the time spent in house, and reducing mental concerns, were mentioned by fathers as the factors influencing their participation in family.

One of the challenges faced by men in playing their fatherhood roles is the lack of a proper role model and a well-defined clear role (Brownson, & Gilbert, 2002). The support provided by the significant others, namely wives, families and friends and formation of men’s support groups can play a key role in providing support and proper role models to fathers that unfortunately are neglected (Carneiro et al., 2012). Recent studies also focused on intersectional relation among work, family and fatherhood (Graham, 2007) and emphasis that workplace can be a unique means of supporting fathers in playing their fatherhood roles more effectively by providing financial support and flexible working hours (Barenski, 2010).

The lack of willingness of some Iranian fathers to take part in this study was one of the research constraints because those men could have different lived experience, cultural meaning and understanding of fatherhood. Notwithstanding this limitation, the study findings offer valuable insight into the lived experiences and socio-culturally constructed reality of fatherhood among Iranian first-time fathers.

## 5. Conclusion

Results of this phenomenological study revealed that in line with global changes regarding fatherhood, as asserted by some scholars including Inhorn and her colleagues (Inhorn et al., 2014). Iranian men’s lived experience and understanding of fatherhood and parenthood, has also changed (Tremayne, 2014). That is, Iranian fathers not only commit to traditional roles, but also accept some new roles such as accessibility for children, providing emotional support and taking part in family affairs. However, it seems there is still a long way to go or to transform Iranian men into actual participant fathers. Lack of paternal role model, stereotypic gender norms about parental roles, lack of adequate knowledge and skills related to child care, long working hours, and mental distress are factors that can have adverse effects on the suitable performance of fathers. It is expected by providing adequate knowledge, skills, and supports we can support fathers to play their social roles and duties more easily and to participate in child rearing more actively in the context of Islamic Republic of Iran.
